# Cytokine Gene Polymorphisms support diagnostic monitoring of Romanian Multiple Myeloma patients

**Published:** 2011-08-25

**Authors:** C Banu, A Moise, CV Arion, D Coriu, A T̆nase, I Constantinescu

**Affiliations:** * National Institute for Medical Assessment and Work Capacity Rehabilitation, BucharestRomania; ** Centre for Immunogenetics, Fundeni Clinical Institute, BucharestRomania; *** Pediatrics Department, Fundeni Clinical Institute, BucharestRomania; **** Centre for Hematology and Bone Marrow Transplant, Fundeni Clinical Institute, Bucharest Romania

**Keywords:** multiple myeloma, cytokine gene polymorphism, interleukins

## Abstract

**Introduction**: cytokines and their receptor genes are very polymorphic. SNPs in the promotor region of the gene may influence the rate of cytokine secretion and may affect the biological activity of the encoded cytokine. A number of cytokines and cytokine receptors have been directly linked to the development of human cancers. The aim of our study was to determine the cytokine gene polymorphism in Romanian multiple myeloma patients.

**Material and methods**: cytokine genotyping was performed in 80 patients and 100 healthy blood donors using molecular biology methods (SSP–Invitrogen, USA).

**Results**: analyzing each polymorphic site, there was an increased frequency of the following genotypes in patients compared to control group: Interleukin–1beta (IL–1β) pos.+3962 TT, IL–12 pos.–1188 CC, gamma–Interferon (γ–IFN) pos.+874 AA, Transforming Growth Factor– beta1 (TGF– β1) codon10 TT, IL–2 pos.–330 TG and pos.+166 TT, Interleukin–4Receptor alpha (IL–4Rα) pos.–33 TC, IL–10 pos.–1082 GG and pos.–592 CC, IL𢀓6 pos.–174 GG. It should be noted that almost one third of multiple myeloma patients had IL–6 pos.–174 GG genotype and 62% IL–10 GCC haplotype. These identified haplotypes are high interleukins producer, and this fact was confirmed by serum IL–6 and IL–10 levels performed by ELISA and enhanced chemiluminiscence methods.

**Conclusion**: these markers could be successfully used, together with other specific clinical and biological parameters, as reliable individualized prognostic factors in multiple myeloma patients.

## Introduction

The cytokines and their receptor genes are very polymorphic. Single nucleotide polymorphisms (SNPs) in the promotor region of the gene may influence the rate of cytokine secretion and may affect the biological activity of the encoded cytokines. A number of cytokines and cytokine receptors have been directly linked to the development of human cancers [1]. 

Multiple myeloma (MM) is a malignancy arising from post–germinal mature B cells characterized by an excess of monotypic plasma cells in the bone marrow secreting monoclonal immunoglobulins in the serum and/or urine, with a concomitant decrease in normal immunoglobulins and lytic bone lesions. Multiple myeloma is a clonal plasma cell neoplasm, which remains incurable despite conventional therapy. MM cells are predominantly localized in bone marrow (BM), and their interaction with bone marrow stromal cells (BMSCs) stimulates transcription and secretion of cytokines from BMSCs. Cytokines in turn not only promote the growth and survival of MM cells, but also reduce the efficacy of conventional drugs [2].

Plasma cells may produce Interleukin–6 (ILx2013;6) by an autocrine mechanism whereas a paracrine mechanism is believed to be involved in the production of IL–6 by bone marrow stromal cells through an interaction between adhesion molecules present on myeloma plasma cells and their respective receptors that are present on bone marrow stromal cells [3]. In addition, control over production of IL–6 may be exerted by other interleukins such as Interleukin–1beta (IL–1β) and IL–10 [4]. Among target cells, the growth of normal and myeloma plasma cells is supported by IL–6, which also induces the differentiation of myeloma plasmablastic cells into mature plasma cells.

Interleukin (IL)–10 is a critical cytokine involved in the terminal differentiation of B cells into plasma cells, but is also a growth factor of malignant plasma cells, produced by myeloma cells from about half the patients and is detected in the plasma of patients with plasma cell leukemia or solitary plasmacytoma. The myeloma cell growth activity of IL–10 is mediated through a gp130 cytokine, oncostatin M (OSM) that is frequently produced by myeloma cells [5].

**The aim** of our study was to determine the cytokine gene polymorphism in Romanian multiple myeloma patients and if there is any correlation between certain genotypes, serum levels of cytokines and disease evolution and prognosis. 

## Material and methods

Eighty patients (F/M: 39/41) diagnosed with multiple myeloma and treated in Fundeni Clinical Institute, Department of Hematology between 2007 and 2011, and 100 healthy blood donors were included in this study. Mean age of the patients was 59 years (36 – 79 years).  

The clinical manifestations of the onset of disease were highly variable, bone pain, secondary anemia and extramedullary infiltration being on the top (Table 1).

Depending on the type of secreted immunoglobulin, distribution in the study group was the following (Table 2).

Most patients were in advanced stages of the disease (Table 3).

DNA was extracted from the whole peripheral blood by using Qiagen reagents. Cytokine genotyping was performed by Invitrogen^TM^ Cytokine Genotyping Kit. The Invitrogen^TM^ Cytokine Genotyping Kit is a PCR–based method designed to detect polymorphisms of thirteen cytokine genes. The kit consists of various formulations of lyophilized primer mixes that are used to amplify genomic DNA by using a 96 well thermal tray. After cycling is complete, the PCR products are loaded onto a 2% agarose gel for electrophoresis and then were visualized under UV illumination and documented by photography. 

**Table 1:** Clinical manifestations of disease onset

Clinical manifestations	Number of patients	Frequency (%)
Pain and bone lysis	59	73,75
Secondary anemia	45	56,25
Extramedullary infiltration	12	15
Chronic renal failure	12	15
Hemorrhagic syndrome	4	5
Hyperviscosity syndrome	2	2,5
Infectious syndrome	1	1,25
Amyloidosis	1	1,25
Smoldering	1	1,25

**Table 2:** Multiple myeloma type

Disease type	Number of patients	Frequency (%)
Immunoglobulin G (IgG)	46	57,5
Immunoglobulin A (IgA)	23	28,75
Micromolecular k	5	6,25
Micromolecular λ	3	3,75
Non–secretory	3	3,75

**Table 3:** Stages of disease

Stages of disease	Number of patients	Frequency (%)
Stage II A	30	37,5
Stage II B	4	5
Stage III A	33	41,25
Stage III B	14	17,5

In addition, to have a complete overview of the patients' immunological status we have analyzed the association of cytokine gene polymorphisms with serum levels of cytokines (IL–6 and IL–10) measured by enhanced chemiluminescence (Immulite). 

The Immulite System assay uses specific antibody or antigen, coated plastic beads as the solid phase, alkaline phosphatase labeled reagent and a chemiluminiscent enzyme substrate. After incubating the sample with the alkaline phosphatase reagent, the liquid reaction mixture, in the Test Unit, is rapidly separated from the bead when the bead is washed, and the Test Unit is spinned at a high speed on its vertical axis. The bead is left with no residual, unbound label. The bound label is then quantitated with a dioxetane substrate, which produces light. A photomultiplier tube detects light emission and the specific designated software generates printed reports for each sample.

## Results

**Figure 1:**
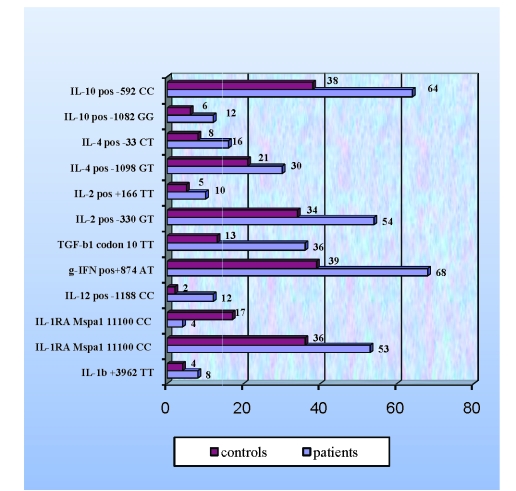
Cytokines polymorphism frequency in patients and controls

Frequency assessment of each polymorphic site, in patients and control group, revealed significant differences in the following cytokines (Table 4, Figure 1)

**Figure 2:**
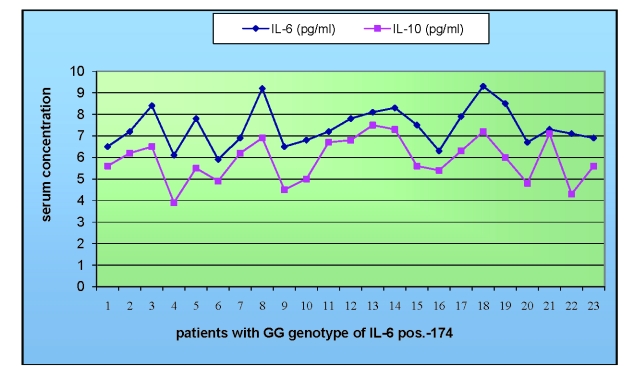
IL–6 and IL–10 serum levels in patients with GG genotype in position –174 of IL–6

It should be noted that 23 (28,75%) of multiple myeloma patients (of which only 2 in the early stages of disease) had IL–6 pos.–174 GG genotype and this genotype was associated with increased serum levels of IL–6 (Figure 2). 

In addition, 17 of these patients also showed increased serum levels of IL–10, and these values were well correlated with the high producer IL–10 – GCC haplotype. Moreover, it should be noted that 64% of all patients had this haplotype, while in controls the frequency was of only 38%.

**Table 4:** Cytokines polymorphism assessment

			Patients (n=80)		Controls (n=100)	
	Position	Genotypes	No.	%	No.	%
IL–1β	+3962	TT	7	8	4	4
IL–1RA	mspa1 11100	CC, CT	3, 42	4, 53	17, 36	17, 36
IL–12	–1188	CC	10	12	2	2
γ–IFN	+874	AT	54	68	39	39
TGF–β1	Codon 10	TT	29	36	13	13
IL–2	–330	GT	43	54	34	34
IL–2	–330	GT	43	54	34	34
IL–2	–330	GT	43	54	34	34
IL–2	–330	GT	43	54	34	34
IL–2	–330	GT	43	54	34	34
IL–2	–330	GT	43	54	34	34
IL–2	–330	GT	43	54	34	34
IL–2	–330	GT	43	54	34	34
IL–2	–330	GT	43	54	34	34
IL–2	–330	GT	43	54	34	34
IL–2	–330	GT	43	54	34	34
IL–2	–330	GT	43	54	34	34
IL–2	–330	GT	43	54	34	34
	+166	TT	8	10	5	5
IL–4	–1098	GT	24	30	21	21
	–33	CT	14	16	8	8
IL–10	–1082	GG	10	12	6	6
	–592	CC	51	64	38	38

## Discussions

Cytokines play a crucial role in the regulation of key pathways of immunity, the balance between cell–mediated (Th1) and humoral (Th2) responsiveness. Genetic variation is common across the human genome. It is estimated that there are more than 7 million single nucleotide polymorphisms (SNPs) with a minor allele frequency of 5% to 10%. Although most SNPs are not functionally important, there is a subset of variants that alter the expression or function of a gene product. Several studies have shown a link between polymorphisms in IL–1β, IL–6, IL–10 and cancer risk. They may also be central to the pathogenesis of MM [7–9].

The interaction between the myeloma cell and the BM microenvironment is central to the growth and survival of myeloma cells. The myeloma cell adheres to the bone marrow (BM) microenvironment, and thereby stimulates angiogenesis and enhances the stimulation of cytokines such as IL–1β, IL–6 and IL–10 [10].

In this study, we address the importance of the polymorphisms  IL–6 GC–174, IL–10  G–1082, C–819, C– 592,  which are known to affect the transcription levels of the genes and are also known to be important for cancer cell growth.

There was no significant difference between patients and the control group on IL–6 –174 GG genotype frequency, but patients who showed this genotype, accompanied by increased serum levels of IL–6, had severe disease needing aggressive chemotherapy

On the other hand, IL–10 –1082GG genotype, was identified as being associated with a better overall survival of patients, together with the favorable prognostic factors.

During the last decade, new treatments with immunomodulatory drugs and proteosome inhibitors have been introduced and have improved the patients' survival. These drugs induce apoptosis of myeloma cells, interrupt the interaction between myeloma cells and stromal cells in the BM, inhibit angiogenesis and inhibit the secretion of IL–1β and IL–6. The treatment effects of the new drugs support the importance of these key regulators of the immune response in the pathogenesis of MM. Inborn genetic variation in these genes may therefore be important for the risk, prognosis and treatment outcome of MM.

## Conclusions

Our findings showed that in almost one third of the studied patients had IL–6 pos.–174 GG genotype and was associated with clinical and biological worsening of MM. IL–6 pos.–174 GG genotype was correlated with increased serum levels of IL–6 in some of the patients and in others with significantly high levels of IL–10.

64% of the multiple myeloma patients had IL–10 – GCC haplotype compare to the controls.

We conclude that these markers could be successfully used, together with other specific clinical and biological parameters, as reliable individualized prognostic factors in multiple myeloma patients
